# New Dental Remedies

**Published:** 1904-06

**Authors:** Alphonso Irwin


### NEW DENTAL REMEDIES.

By Alphonso Irwin, D. D. S.

Report of Committee on Materia Medica, New Jersey State Society.

In consequence of dentists devoting their energies more to the art than the science of dentistry dental materia medica is still in the evolutionary stage. A thorough and positive knowledge of medicine will transform the dental artisan into a specialist of medicine.

If you believe dentistry is a specialty of medicine, scientific knowledge concerning the action of drugs in diseased conditions of the mouth and teeth is essential.

If you do *not* believe dentistry is a specialty of medicine, you are aware that no dentist can relieve suffering who ignores scientific medication. Whosoever regards it as unimportant is bound to be tripped up sooner or later in his professional career, to his mortification and shame as well as loss of prestige and fees.

About 350 new remedies are discovered annually. Many new remedies announced one year are supplanted by the remedy with a corresponding therapeutic action discovered the following year. There has been a slow but decided revulsion of feeling against the efforts of enthusiasts to advance the claims for therapeutic efficacy of new chemicals. This caution has not prevented experimental investigation in practical medication. On the contrary, the judicious acceptance of that which is good and the rejection of worthless remedies has naturally followed.

We can state as a result of a correspondence with dentists, physicians, editors and college professors living in all parts of the

United States and involving the sending of over 200 letters upon the subject that *no* strictly *new* remedy of wonderful value in dental practice has been discovered during the past year.

There have been produced, however, new uses for drugs of recent introduction, new combinations of old drugs with different therapeutic effect in pathological conditions from that of the individual constituents and new uses discovered for old remedies hitherto unpublished to the profession.

The remedies which are of the greatest interest to the dental profession according to the allopathic classification are: Anæsthetics—general and local, analgesics, antiphlogistics, antiseptics, disinfectants, hemostatics, germicides, laxatives, nervines, stimulants, tonics.

Airol (Bismuth Oxy-Iodo-gallate) is an antiseptic which I found popular upon the continent three years ago. It has been commented upon occasionally in the United States during the past year. It is a good substitute for iodoform and possesses the advantages of being non-toxic, odorless and non-irritant—properties which commend it to the dentist wherever iodoform is advantageous. I prefer aristol. In general surgery in Russia airol has been used very successfully in the army in comparison alternately with iodoform on 200 soldiers.

Septoforma is a product of formaldehyde. According to Engels, a three per cent. solution destroys staphylococcus pyogenes aureus in three minutes, the cholera vibrio in one minute, and the typhoid bacillus in ten minutes. Instruments of various metals exposed for three days to thirty per cent. solution of septoforma, and none of them showed the slightest change except those made of aluminum. It seems to have advantages over other similar disinfectants, owing to a much less penetrating odor, absence of local irritation or injury to metal instruments, and that it has a considerable degree of germicidal power. It is recommended for the disinfection of instruments in the strength of five per cent. to ten per cent. solution and as a wash for wounds in a three per cent. solution.

Adrenalin was discovered and introduced by Jokichi Takanimi, who read a paper before you at our last meeting upon my invitation.

Adrenalin has claimed much attention during the past year as an agent for the painless extirpation of the pulp, as an obtundent for sensitive dentine, as a local anæsthetic and hemostatic. (Items of Interest, June, 1903, page 401, by W. Clyde Davis, M. D., D. D. S.)

Positive results are claimed from its application for painless pulp extirpation when the following method is followed: Apply the rubber dam, dry the cavity. If the pulp is covered by softened dentine apply one drop of adrenalin, then one drop of a forty per cent. solution of formaldehyde. If quite a distance from the pulp use slight but continued pressure with a soft rubber plug for a few seconds. Excavate as near to the pulp as possible.

Apply a second crop of adrenalin. Lay in the cavity a few crystals of cocain, apply one drop of the forty per cent. solution of formaldehyde. Apply pressure with the rubber plug first slightly, gradually increasing the pressure until at the end of one minute you can knead the rubber in the cavity with burnishers without inflicting pain. If upon running a nerve broach slowly up to the apex of the root there is pain, repeat the application. Then you can remove the pulp painlessly. Where there is excessive hemorrhage apply adrenalin under pressure for fifteen seconds. Insert non-irritant dressing for twenty-four hours and fill. If indications are favorable, fill immediately. The tendency to elongation and soreness of the root is diminished, the peridental membrane in many cases losing "tactile sense" where adrenalin is used.

The advantages claimed are: Adrenalin is painless; a time saver; tooth discoloration does not occur; after soreness is slight and many times wanting; lastly, your application is a powerful antiseptic.

Dr. D. W. Barker says (in a paper read before the Second District Dental Society of New York, Dec., 1902: "Its discoverer, Dr. W. H. Birchmore, so designates a new disinfectant which combines bromine and chlorine in a new way. It is not a preparation of a chloride of lime. Nascent bromine and chlorine do the work of disinfection. Lime is the connecting chemical. Water will dissolve more than

D-5559

its own weight of bromo-cloron. It is unstable and breaks up upon contact with the faintest trace of a foreign acid. The lime combines with the acid. The bromine and chlorine are freed to destroy any bacteria or animal matter present." Either directly or by the formation of nascent oxygen. *The solution* has *not* the *least effect* upon *living animal membranes.* The chief claim for its use lies in the fact that its action is confined to dead material. It never attacks the teeth, lips and gums. Yet it destroys dead material and bacteria. This new reagent has every advantage of all other reagents and disinfectants without any of their defects.

In treating abscess you open and cleanse root canals with bromo-chloron, flushing out the abscess thoroughly through the fistula with bromo-chloron. Where practicable discharging several syringefuls into it until not a trace of pus remains. No precautions need be taken to protect the mouth.

Dr. Barker claims bromo-chlorin is as powerful a germicide as bichloride of mercury.

Ecthol is an antiseptic used internally as well as externally. The following experience with ecthol is recorded in a case of septic poisoning after extraction: G. S. F., aged eleven, had tooth extracted June 20, after a preliminary hypodermic injection of a four per cent. solution of cocaine. On July 4 she began to complain of tender gums and neuralgic pains of head and face. Upon examination the tissues surrounding the cavity left by tooth were found ulcerated and inflamed and covered by a dirty greyish slough. The surrounding teeth were tender and gums boggy and engorged with blood. Temperature 103° F. Tongue coated. Pulse 120. Loss of appetite, and patient had a severe chill once in twenty-four hours, followed by exhausting sweats. Swabbed cavity and surrounding tissues every three hours with pure ecthol. Gave saline purges and one-drachm doses of ecthol well diluted with water every four hours. Improvement was noticed on third day of treatment. Fever, sweats and rapid pulse were controlled. The unhealthy granulations disappeared and convalescence was established in ten days. Undoubtedly the symptoms in above cases were produced by the presence and absorption of septic material, and each surgical procedures were refused. I would not wish to be understood as taking a stand against surgery in cases were an operation is unavoidable, but I do believe that ecthol in some way is antagonistic to the chemical exudates produced by bacteria and is worthy of an extended trial at the hands of the medical profession.—Edmond J. Melville, M. B. C. M., Bakersfield, Vermont, in *Medical Brief.*

Dr. M. J. Reboul (*Revue médicale*) has used ethyl chlorid as a general anæsthetic in more than five hundred cases. He states that by means of his agent anæsthesia is easily induced, and that patients do not experience any unpleasant sensation. It should be given in small doses, 5 c.c. every five minutes, and the total quantity administered should not be greater than 25 c.c. In children and old people the dose should be half the quantity recommended for the average adult.

The administration by means of a compress is the simplest and most convenient method. It is recommended that air should be excluded. The transmission of an intensely cold sensation to the hands of the operator from the evaporation of the ethyl chlorid in the compress occurs simultaneously with the beginning of the anaesthesia; two or three minutes afterward the anaesthesia is complete. The anaesthesia disappears upon the removal of the compress; but this period is followed by one of analgesia, during which, although at this time the patient is not fully conscious, surgical operations can be performed without his being aware of it.

Dr. Reboul has not observed any unfavorable symptoms consequent upon ethyl chlorid.

An ethyllic compound called narcotile promises to become a formidable competitor of nitrous oxide gas as an asaesthetic for the painless extraction of teeth and other dental operations where general anaesthesia is desirable. The value of chloride of ethyl as a local anaesthetic has been long known and the bromide of ethyl has been used as an anaesthetic, but the introduction of an ethyllic compound for producing general anaesthesia has been of more recent date. It appeared in England in April, 1901, and later in the United States.

The advantages claimed for narcotile are its "unalterable composition, rapidity of action, harmless and easy employment." Among the thousands of cases where narcotile has been administered, its application has never been attended with accident. In producing general anaesthesia with narcotile, two hours after the last meal should elapse, the patient must be free from any constricting clothing around the waist or neck, and placed in a position as near horizontal as possible. All artificial teeth must be removed, and if for extraction the gag must be applied before narcosis.

Sixty-seven c.c. (mililitre = 16.2318 m., about 113 m.) is sufficient to cause anaesthesia in from half to one minute and lasting from five to ten minutes. By repeating or prolonging thea dministration of narcotile anaesthesia may be prolonged without inconvenience from ten to fifty minutes.

The general effects of narcosis produced by narcotile nearly resembles those of sulphuric ether. Much more speedy than chloroform or nitrous oxide gas, narcotile acts upon the sensory nerves and spinal centers, then upon the motor centers and motor nerves, and finally upon the medula oblongata. Complete anaesthesia is so rapid that the different stages of narcosis can hardly be noticed. There are very seldom signs of excitement at the first stage of its application. The condition of complete anaesthesia may be recognized by the moderation of the pulse and the respiration. The patient lies quietly as if in a deep sleep; the movements of respiration are regular and rather less frequent than in normal sleep. The patient inspires deeply, and sometimes is snoring. The pulse becomes full and soft and scarcely accelerated, sometimes even slower than at the commencement of inhalation. The muscular system is not entirely relaxed except in children. The best way to ascertain the fact that anaesthesia is complete is to raise the arm of the patient and then allow it to drop by his side. If the stage of complete anaesthesia has been reached, the limb will fall like an inert mass. If any degree of rigidity persist, a fact which may be readily ascertained by flexing the arm at the elbow joint, the patient is not fully under anaesthesia.

Sensibility may also be tested by pinching the skin sharply between the nails of the finger and thumb. The indications thus obtained are more reliable than the results of touching the conjunctiva of the eye.

Consciousness returns very quickly; the patient does not experience any period of stupor or coma, which usually follows the application of other anaesthetics.

Narcotile, acting chiefly on the cerebro-spinal centers and on the motor nerves, does not affect the heart, but we should advise the surgeon to make inquiries as regard undue indulgence in alcohol, or if the patient is undergoing treatment for cardiac disease, these cases being a contra indication for the use of "narcotile."

The digestive properties of papoid have been the subject of investigation by chemists, and its power and value have been proved beyond a doubt.

An aqueous solution of papoid soon spoils, but a glycerine solution will not readily ferment. A glycerine solution is particularly employed for dissolving false membrane in the throat in diphtheria and in cleansing of foul wounds in surgical practice. Any of the usual chemical antiseptic agents may be prescribed with papoid. Where it is desired to increase its activity, the addition of five grains of sodium bicarbonate to each 100 minims of the solution is a decided advantage. It acts at any temperature, but when possible, as in surgical cases and certain forms of skin disease, it is advised that the part to which it is applied should be kept warm with hot cloths or some other means, as a moderately elevated temperature favors its action.

It is claimed that papoid, prepared by Johnson & Johnson, is a preparation superior to papin, which Dr. Harlan reported upon so favorably in the dental journals.

*Recommended for Trial.*

It is suggested in addition to those remedies already mentioned a careful trial and a tabulated record of the following drugs be made and reported upon at a future time:

*Antiseptol*—An antiseptic for internal and external administration.

*Acetozone*—A powerful germicide.

*Alka-thymal*—Antiseptic and detergent. Used in pyorrhoea and as a mouth wash.

*Aromatic Tincture of Red Gum*—Soluble in water or alcohol. An astringent mouth wash, styptic for hemorrhage after dental operations.

*Alka-thyptol*—A preparation of thymol and eucalyptus, useful in the treatment of oral catarrah, chronic nasal catarrh and diseased mucous surfaces.

*Enzymol*—A deodorizer, a solvent of septic naterials, potent, painless, harmless; has a remarkable healing curative effect. Dilute with an equal volume of water. Use for abscess, necrosed bone, gangrenous pulp, pyorrhea alveolaris, disease of antrum. Highly recommended by Drs. T. R. Chambers, R. T. Morris and J. W. Glitsman in treating mucous surfaces of eye, ear, nose, throat and mouth.

(Lithium Bitartrate Salicylic Acid and Sodium Bitartrate) and

*Lithos*—Uric acid solvents for internal administration in the systemic treatment of pyorrhœa alveolaris (caused by Lithunia).

*Nargol* — A new silver compound. Solutions injected for disease of the antrum and along pus tracts. Its use is suggested for treatment of putrescent pulps and sensitive dentine in the molars.

*Phenoline*—An antiseptic disinfectant and germicide. Used as a mouth wash and in the treatment of catarrh; for wounds, scalds and burns. Dilute with two to ten parts of water.

*Olecco*—A pleasant laxative antacid, used in constipation; the anti-pholgestic treatment required in conjunction with local treatment of disease of the mouth and teeth. Recommended highly by Dr. L. S. McMurtie, Louisville, Ky., and Dr. Veschner, Chicago, Ill.

*Pyrozone*—Prepared according to the standard of the U. S. Pharmacopœia. Not only maintains its reputation but its use is on the increase.

*Ut-A-Sol*—For neuralgia and rheumatic pains; dose 10 to 20 grains; total dosage limit per 24 hours one to three ounces. Is not a depressant but tonic. It is an internal antiseptic. Sterilizes urine.

*"Solidified Phenol"*—A convenient and efficient application for pulpitis.

*"White Enameled Glycerine Proof Cases for Anhydrous 95 per cent Glycerine Suppositories"*—A convenient and efficient laxative.

*Glyco-Thymoline Applicator* (Kress & Owen) designed by E. C. Rice, of Philadelphia, consisting of a rubber ball and tube conveniently shaped to inject fluids into pus pockets, abcesses, fistulas, which intelligent patients can take home and use.

The Oakland Chemical Co. also have a small syringe designed for injecting remedies into contact with diseased surfaces. They are exceedingly useful and the subject of new remedies would not be complete without mention of the new applicators for using the drugs in dental medication.

*Text Books* on Dental Materia Medica. Lea Bros. & Co. have issued an edition of Long's Dental Materia Medica.

P. Blakiston Son & Co. have published a new revised and enlarged edition of "Gorgas' Dental Medicine" (7th ed. by F. J. S. Gorgas, M. D., D. D. S., Baltimore, Univ. of Med.).

In conclusion, remedies recently introduced possessing more or less merit, have been necessarily omited because it would require volumes to enumerate them and their action in detail.—*Items of Interest.*

